# Texas Quarantine Expenses

**Published:** 1895-01

**Authors:** 


TEXAS QUARANTINE EXPENSES.—The total expense of administering quarantine for the four years of Governor Hogg's administration, Dr. Swearingen, State Health Officer, has averaged $45,000 a year, less cost of repairs, etc., for 1893-4; and $35,000 special appropriations has been expended for new stations, and equipments and repairs. In no year have the expenditures exceeded the appropriation; and excepting the State Health

Officer and the officer at Galveston and at Velasco,—the former necessarily on duty the entire year, the Galveston officer on duty during the winter after close of quarantine season, on account of cholera in Europe the past two years, and the Velasco officer on duty at half pay after close of season,—the pay of no one officer has exceeded, in any one year, $1800.

Considering the extensive coast line to be guarded, and the extensive frontier border on the Rio Grande, which it is necessary to guard against small-pox throughout the year, this is a remarkable showing. Six gulf coast quarantine stations, and three frontier stations, necessarily in full operation from May to November, making nine stations; and two gulf and the three border stations in operation throughout the year, making five; and in addition to this, during the cholera scare in '92-3 it was necessary to establish and equip four new stations along the north and east State line. These were officered, and their pay, as well as the cost of equipment, had to come out of the regular appropriation, as no provision had been made for, and no prudence or forethought could have anticipated, such contingency. When we reflect, then, that thirteen (sometimes fourteen) quarantine stations were smoothly and successfully conducted on $45,000 a year, less the cost of equipment of new and repairs of other stations, for which no extra provision was made in the appropriation of '93-4, the JOURNAL thinks Texas has cause for congratulation.

